# Addressing loneliness and social isolation through the involvement of primary and secondary informal caregivers in nursing homes: a scoping review

**DOI:** 10.1186/s12877-024-05156-1

**Published:** 2024-06-25

**Authors:** Dominique Autschbach, Anika Hagedorn, Margareta Halek

**Affiliations:** School of Nursing Science, Witten/Herdecke University, Alfred-Herrhausen-Straße 50, 58455 Witten, Germany

**Keywords:** Nursing home, Involvement, Psychosocial interventions, Informal caregivers, Loneliness, Social isolation, Scoping review

## Abstract

**Objectives:**

To clarify the mechanisms of interventions addressing loneliness and social isolation in older adults living in nursing homes through the involvement of primary and secondary informal caregivers.

**Methods:**

This scoping review was performed by two independent reviewers, covering the period between 2011 and 2022 and the databases MEDLINE, CINAHL, PsycINFO and Scopus. It included terms related to **(**A) informal caregivers, (B) nursing homes, (C) psychosocial interventions, (D) involvement and (E) social isolation or loneliness.

**Results:**

Thirty-three studies met the inclusion criteria. Although there were various definitions and assessment tools related to social isolation and loneliness, the studies referred to three dimensions of these concepts in nursing home residents: the quantity of social interactions, the perception of these encounters and biographical changes in social relationships. Most studies did not explicate the mechanisms of these interventions. The review uncovered the following aspects of intervention mechanisms: increasing opportunities for social contact, creating meaningful encounters, maintaining existing relationships with primary informal caregivers and establishing new ones with secondary informal caregivers.

**Conclusion:**

Studies reporting on interventions addressing loneliness and social isolation in nursing home residents need to clarify and detail their intervention mechanisms in order to foster more targeted interventions. In addition, there is a need for further research on large-scale programs or care philosophies in this field and the development of intervention designs, which allow for tailored intervention formats in order to respond to the individual perception of social relationships.

## Background

Loneliness and social isolation are mayor challenges in geriatric care. For the population over 80 years of age it is estimated the prevalence rate of loneliness lies between 27.1% for severe and 32.1% for moderate cases, while it is at 33.6% for social isolation [1]. It is estimated the mean prevalence of loneliness in nursing home residents is 61% for moderate and 35% for severe levels [2]. These alarming overall tendencies were highlighted by the COVID-19 pandemic and its aftermath [3–6]. The health effects of loneliness and social isolation further underline the concern: Both states are generally linked to increased mortality [7]. In addition, studies on older adults living in long-term care settings report that loneliness increases the risk for depression, suicidal ideation and frailty [8]. There are various risk factors for social isolation in nursing homes: reaching from individual health related communication barriers to systemic issues, such as the location of many facilities, and structural challenges including socioeconomic disadvantages or discriminatory public perceptions [9].

Frequently social isolation is defined as the ‘objective’ number of social interactions, while loneliness characterizes the ‘subjective’ evaluation of these interactions [10]. However, there is considerable variation in how both concepts are defined and operationalized [11–13]. Thus the term social isolation touches upon various aspects of the number of social contacts and subjective evaluations of these encounters [12]. Similarly each definition of loneliness emphasizes a specific aspects of the phenomenon, for example highlighting the cognitive evaluation of the actual and desired relationships or the experience of a situation as lacking social relationships [13]. Robert Weiss’ seminal work on loneliness distinguishes between emotional and social loneliness – the former referring to the lack of intimate relationships while the latter describes the feeling of missing a social network [14]. Building on this distinction a recent conceptual review on loneliness in adults differentiates between three dimensions of loneliness: the social dimension of loneliness highlights social connections and the feeling being socially isolated, the emotional dimension touches upon the quality of relationships, and the existential dimension of loneliness captures the feeling of being fundamentally (and not only temporarily) separated from others [15].

For older adults living in nursing homes, functional relationships with staff alone may not compensate for loneliness and social isolation. Contrary to community dwelling older adults, nursing home residents can potentially profit from the company other residents [16]. A crucial aspect for nursing home residents is support from family members and friends [17, 18]. In addition, older adults living in nursing homes can receive support from a range of external community actors, voluntary workers or institutions like schools and associations, providing various forms of informal care and companionship. Though most of the literature refers to informal caregivers as family members or friends, there are concurring understandings of the term [19]. In the context of nursing homes, we distinguish two main groups: *primary informal caregivers* who have a biographical relationship with one nursing home resident (e.g. relatives, friends) and s*econdary informal caregivers* who have a connection to the nursing home itself (e.g. schoolchildren, members of associations, voluntary workers). Secondary caregivers play a robust role in expanding the circle of social contacts for residents [20–22] and opening the nursing home to the larger community. The inclusion of these groups in the nursing home is a complex process [23] involving informal caregivers’ access to everyday activities and active engagement in decision-making and the care process.

There are various psychosocial interventions addressing the involvement of primary and secondary informal caregivers in nursing homes, these interventions change the behaviour of these actors with the aim to advance or retain the mental or physical health and well-being of residents. However, the facilitation of this involvement remains underexplored [24]. There are increasing calls for refining and tailoring interventions against loneliness and social isolation for individuals and specific groups of older adults [10, 25]. Previous reviews on interventions against loneliness highlight the specificity of interventions for older adults in general [26, 27] or nursing home residents in particular [28, 29], we add to this debate by looking at a sub-set of these interventions – interventions involving informal caregivers into the facility. This review therefore explores the field of intervention studies addressing loneliness and social isolation through the involvement of both groups of informal caregivers in nursing homes. It aims to (A) clarify how intervention studies employ the concepts of loneliness and social isolation and (B) subsequently lay out the mechanisms through which interventions address the dimensions of loneliness and social isolation. Mechanisms describe how an intervention brings about a change in a certain outcome [30]. The review hence contributes to the endeavour to broaden theoretical understandings of how interventions contribute to reducing loneliness and social isolation [10, 27].

## Methods

As the review explored the broad extent of the knowledge on intervention mechanisms against loneliness and social isolation, we chose a scoping review methodology and based our review on the criteria of the *Joanna Briggs Institute Manual for Evidence Synthesis* [31]. We used a modified version of the PRISMA Extension for Scoping Reviews (PRISMA-ScR) and published a protocol for the review (https://osf.io/vjzqw/). With regard to this protocol, the search had to be limited to empirical studies and conceptual papers to better reconstruct the intervention mechanisms themselves.

### Inclusion criteria

We included studies on *psychosocial interventions* against *loneliness and social isolation*. The term psychosocial interventions refers to a wide array of activities aimed at maintaining or improving the functioning, well-being and social relationships of older adults [32]. These interventions focused on the *involvement* of primary and secondary informal caregivers in the facility life of *nursing homes*, i.e., institutions providing 24-hour support to people requiring assistance during (instrumental) activities of daily living due to identified health needs [33]. Studies comparing nursing homes with hospital or community settings were also included. Hence, the populations covered in this review included nursing home staff, *primary and secondary informal caregivers* and residents of nursing homes, with a focus on primary informal caregivers such as partners, relatives and friends and secondary informal caregivers, i.e. broader community actors engaging with the nursing home. For our review, we define primary informal caregivers as people who feel a sense of belonging towards a certain person within the nursing home and maintain a biographical relationship. Secondary informal caregivers are defined as people that feel a sense of belonging towards the nursing home itself. Studies on nursing home residents alone were included only if the residents were explicitly addressed by the interventions as providers of support to their peers as secondary informal caregivers.

As it was the aim to explore the field of studies, the review covered peer-reviewed articles as well as grey literature, incorporated studies with qualitative as well as quantitative designs regardless of their quality. We excluded letters to the editor, posters, review articles and commentaries and included empirical intervention studies and conceptual papers on interventions in English and German published between 2011 and 2022. Focussing the sample on empirical intervention studies and conceptual papers allowed us to pinpoint the outcomes of these interventions as well as their mechanisms.

### Search strategy

We carried out a preliminary search in MEDLINE and a manual search in PROSPERO to refine the search terms. The search in April 2021 and an update in December 2022 included the following information sources: MEDLINE, CINAHL, PsycINFO and Scopus. The search strategy comprised terms related to (A) informal caregivers, (B) nursing homes, (C) psychosocial interventions, (D) involvement and (E) social isolation or loneliness (Table 1). We hand-searched for additional relevant sources in the bibliographies of articles included in the full-text screening and carried out a forward citation of these articles in *Google Scholar*.

**Table 1:** Search terms used in PubMed

Subject area	Search terms
Informal carers/caregivers #AND	“famil*“[tiab] OR “family” [mh] OR “child*“[tiab] OR “relative*” [tiab] OR “partner*” [tiab] OR “volunteer*” [tiab] OR “siblings” [mh] OR “spous*” [tiab] OR “spouses” [mh] OR “informal care*” [tiab] OR “communit*” [tiab]
Nursing homes #AND	“long-term care” [tiab] OR “long-term care” [mh] OR “nursing home*” [tiab] OR “nursing homes” [mh] OR “care home*” [tiab] OR “retirement home*” [tiab] OR “residential aged care facilit*” [tiab] OR “assisted living facilities” [mh] OR “residential facilities” [mh] OR “residential care” [tiab] OR “housing for the elderly” [tiab]
Psychosocial interventions #AND	“program*” [tiab] OR “intervention*” [tiab] OR “strateg*” [tiab] OR “concept*” [tiab] OR “tool*” [tiab] OR “instrument*” [tiab] OR “care model*” [tiab] OR “implement*” [tiab]
Involvement #AND	“stakeholder participation” [mh] OR “participat*” [tiab] OR “relationship*” [tiab] OR “collaborat*” [tiab] OR “involve*” [tiab] OR “interact*” [tiab] OR “role*” [tiab] OR “engag*” [tiab] OR “cooperat*” [tiab] OR “partnership” [tiab]
Loneliness/social isolation #AND	“social isolation” [mh] OR “isolation” [tiab] OR “solitude” [tiab] OR “solitary” [tiab] OR “loneliness” [mh] OR “loneliness” [tiab] OR “lonely” [tiab] OR “boredom” [tiab] OR “separation” [tiab] OR “separated” [tiab] OR “distancing” [tiab]

### Selection process

**Fig. 1:**
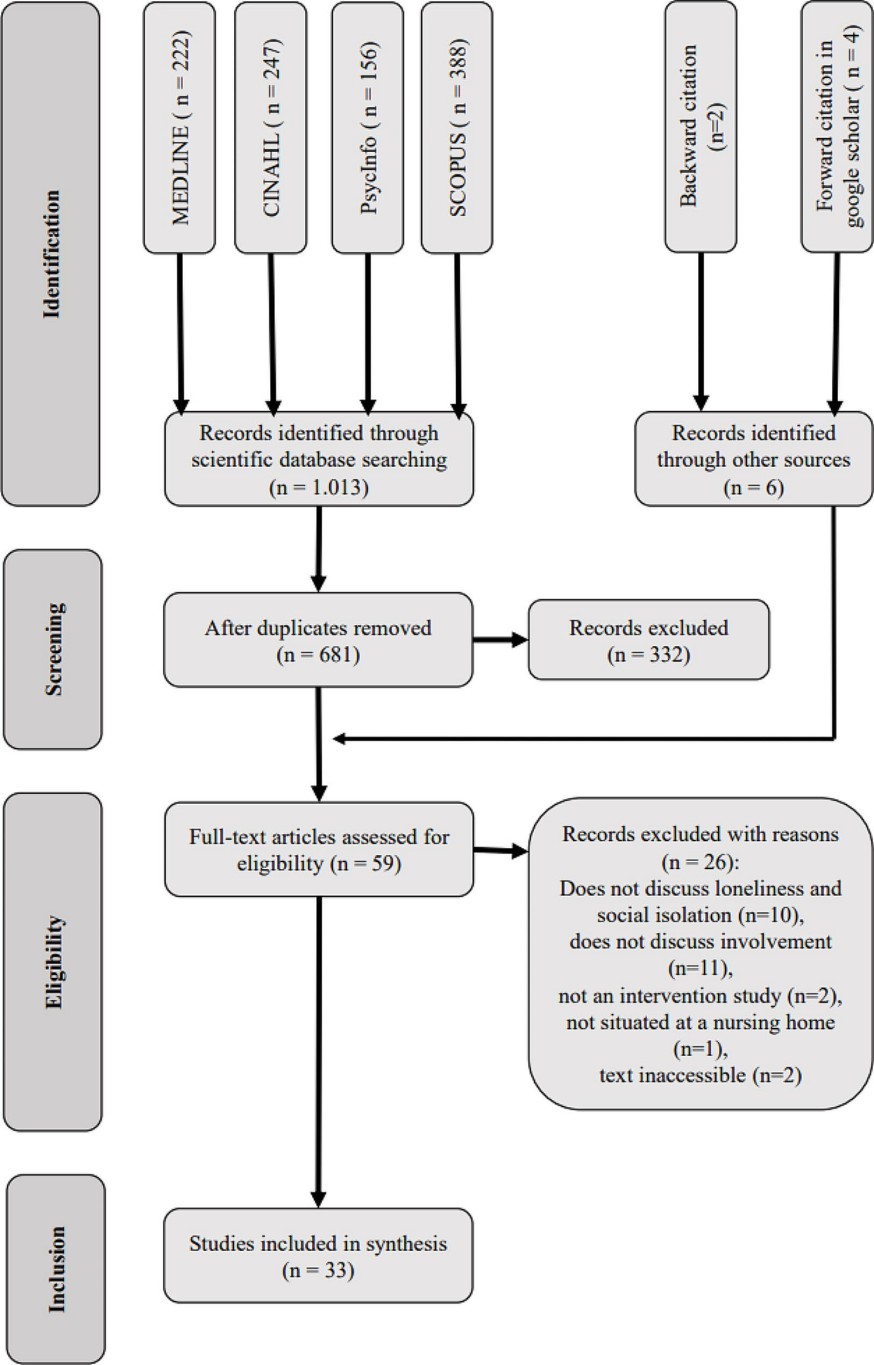
PRISMA Flow Diagram

We gathered the included titles found in different information sources in EndNote 20 (Clarivate Analytics) and removed duplicates from the list (Fig. 1). Afterwards, we screened the titles and abstracts of the remaining articles for their eligibility according to the five inclusion criteria. Two independent reviewers (AH and DA) screened and selected articles and carried out the data extraction, and a third reviewer was consulted in case of divergences (MH).

### Data extraction and analysis

Based on the JBI criteria for data extraction in scoping reviews, we used an extraction sheet with information on the country of intervention, methodology, population included, sample size and type of care facility; a detailed description of the intervention; the definition, use and/or measurement of the concepts loneliness and social isolation and the challenges and facilitators mentioned in the studies; and a bibliographical note.

We started by comparing the definitions, assessments and implicit uses of loneliness and social isolation across articles based on the extraction sheets. Subsequently, we employed these dimensions to the intervention descriptions in order to highlight the implicit intervention mechanisms. In order to clarify the mechanisms of these interventions, we furthermore assessed the description of the mechanism and interpreted included studies with regard to the type of relationship (establish/maintain relationships, meaningful connections) and involvement (group, dyad) they generated.

## Results

As a result of the database search, a total of 1,013 articles were retained (Fig. 1). After removing 332 duplicates, we screened the title and abstract of the remaining 681 articles and added six sources through backward and forward citation. We performed a full-article screening of 59 articles, of which 26 were excluded for various reasons (see Fig. 1), leading to 33 articles included in the review (Table 2).

**Table 2:** Articles included in the review

Author	Year	Country	Sample size	Methods	Type of intervention	Intervention description	Intervention mechanism			Loneliness and/or social isolation	Outcomes
							General mechanism description available	Relationships	Involvement		
Akinbohun	2015	US	staff (*n* = 7)	Semistructured interviews	Combined intervention programme	Components consist of various interventions such as music, humour and reminiscence therapy, social support programmes, animal-assisted therapy, robotic companion animals, elders-helping-elders programs, recreational therapy, information and communication technology, Eden Alternative program	N/A	Maintain relationships, establish relationships, meaningful connections	dyad, group	loneliness	---
Angelou et al.	2022	AU	artists (*n* = 46), staff (*n* = 20)	Group discussions, interviews and reflexive diaries	Intergenerational live-in programme	University students lived at a nursing home and volunteered by spending time with residents or organising group activities	provides opportunities for engagement	establish intergenerational relationships, create meaningful connection	dyad, group	loneliness, social isolation	Reported establishing a community of care and collective based on belonging and shared doing
Barbosa Neves et al.	2017	CA	residents (*n* = 5), informal caregivers (*n* = 5)	Semistructured interviews, assessment tools, field observations, usability and accessibility testing	Tablet-based messaging application	Residents used the application to send text, images or audio data and videocall	increase opportunities for connectedness	maintain family relationship, meaningful connection	dyad	loneliness, social isolation	Reported no changes in loneliness and social isolation
Barbosa Neves et al.	2019	CA	residents (*n* = 12), informal caregivers (*n* = 12)	Semistructured interviews, assessment tools, field observations, usability and accessibility testing	Tablet-based messaging application	Residents used the application to send text, images or audio data and videocall	increase opportunities for connectedness	maintain family relationship, meaningful connection	dyad	loneliness, social isolation	Reported no changes in loneliness and social isolation
Beogo et al.	2021	CA	N/A	Semistructured interviews, focus groups, survey	Web-based platform	Residents of a minority linguistic group can use platform for voice/video calls, text messaging and voice-mailing	support communication needs	maintain family relationship	dyad, group	loneliness, social isolation	---
Brownie & Horstmannshof	2011	N/A	N/A	Conceptual paper	Intervention plan	The intervention plan consists in an assessment, planning, implementation, and evaluation. Suggested interventions include email and social media contact, reminiscence therapy, pet ownership, gardening, leisure and volunteer activities, Eden Alternative managerial changes	N/A	Maintain relationships, establish relationships, meaningful connections	dyad, group	loneliness	---
Brune	2011	N/A	N/A	Conceptual paper	Managerial changes	The Eden Alternative programme includes various managerial changes, such a implementation of a person-centered work, decision-making close to the residents, etc.	N/A	Maintain relationships, establish relationships, meaningful connections	dyad, group	loneliness	---
Cheetu et al.	2022	CA	residents (*n* = 11), staff and volunteers (*n* = 21)	Group discussions, interviews	Music programme	The “Music Care” programme consists in a music care expert initiating music activities and setting up a team at the nursing home. The activities ranged from individual music visits to choir sessions.	N/A	N/A	dyad, group	loneliness, social isolation	---
Chu et al.	2021	CA	residents (*n* = 13), staff and informal caregivers (*n* = 15)	Semistructured interviews	Exergaming system	The MouvMat combines physical exercise with video-gaming. Users played different games through moving on a surface.	N/A	establish relationships to other residents	group	social isolation	---
Cotten et al.	2013	US	residents (*n* = 205)	Assessment tools and standardized questions	Online communication	Residents received training on computer and internet use in order to communicate and search for information.	facilitate communication	maintain relationships with friends and family	N/A	loneliness, social isolation	Reported decreased loneliness levels and an increased quantity of communication but no changes in social isolation.
Evans et al.	2022	GB	artists (*n* = 46), staff (*n* = 20)	Reflexive journals, standardized feedback forms	Creative ageing programme	The programme consisted of 23 separate arts projects, such as dance, drama, music, visual arts and poetry events.	increase opportunities to maintain social network	establish relationships with the community	group	loneliness, social isolation	Claimed that the intervention decreased social isolation and reduced loneliness.
Follmann et al.	2021	DE	residents and hospital patients (*n* = 70)	assessment tools, telephone interviews	Care robot	The care robot Temi was used to carry out videocalls. The robot has a humanoid design, can drive autonomously and residents can control it via voice control. humanoid robot that can be operated by voice control were able to perform video calls	initiate communication	maintain family relationships	Dyad	loneliness, social isolation	Reported no relevant effect on loneliness.
Foster et al.	2021	CA	residents (*n* = 265)	assessment tools, telephone interviews	Music programme	The “Music Care” programme consists in creating a tailored music action plan at the nursing home. The activities ranged from individual music visits to choir sessions.	N/A	N/A	dyad, group	loneliness, social isolation	Reported a decrease in loneliness.
Gurung et al.	2022	AU	student (*n* = 1), residents (*n* = 2), staff/volunteers (*n* = 3)	Semistructured interview	Intergenerational living arrangement	During the “Food for Life – Better Care” programme a university student lived in a nursing home and dedicated 30 h per month to interacting with residents.	increase opportunities for socialisation	establish intergenerational relationships	N/A	loneliness, social isolation	Reported a reciprocal relationship and reduced perceived isolation.
Hoang et al.	2021	CA	N/A	Study protocol	Computer education programme	For the intervention ‘enTECH’ a trained student is supposed to provide support in email communication.	N/A	maintain family relationships, establish intergenerational relationship	Dyad	loneliness, social isolation	---
Horan & Perkinson	2019	US	residents (*n* = 10), students (*n* = 14)	N/A	Intergenerational visiting programme	During the programme university student volunteers visited nursing homes and carried out seasonal activities, such as crafts and decoration.	Create opportunities for social connections	establish intergenerational relationships	Group	social isolation	---
Jones & Ismail	2022	GB	staff and external professional stakeholders (*n* = 49)	Interviews	Intergenerational food-based programme	The programme involved preschool and school children into growing, cooking, eating, and community activities at the nursing home.	intergenerational transmission of values, promoting connections	establish intergenerational relationships	Group	loneliness	Reported reduced perceived loneliness.
McAllister et al.	2020	N/A	residents (*n* = 3), staff and informal caregivers (*n* = 7)	Field notes, focus groups, interviews	Digital reminiscence application	The application “Memory Keeper” gave audiovisual prompts provided by family members to residents with dementia in order to spark reminiscence and meaningful engagement.	stimulate reminiscence, meaningful engagement	maintain family relationships, meaningful connection	Dyad	loneliness	---
Moyle et al.	2014	AU	residents (*n* = 5), informal caregivers (*n* = 6), staff (*n* = 7)	Semistructured interviews, call records, video observational data	Telepresence robot	The robot “Giraff” was used as a videoconferencing tool for residents to communicate with family members. Family members received training material and training sessions.	enhance communication	maintain family relationships	Dyad	social isolation	Reported reduced social isolation and increased connection.
Ngamaba & Heap	2022	GB	N/A	Informal responses	Multicultural singing intervention	The intervention brought performances of a local church choir of African heritage to a nursing home. It included training on the intervention and loneliness for the musicians.	N/A	establish relationships with the community and other residents	Group	loneliness	---
O’Rourke et al.	2021	CA	N/A	Conceptual paper	Integrated music programme	“Music Connects Us” consists in group sessions of improvised music-making for residents with dementia.	foster group engagement, communicate through music	establish relationships with volunteers and other residents	Group	loneliness	---
Peng	2018	AU	residents (*n* = 5)	Interviews, collective work on the prototype	Digital visual storytelling and reminiscence tool	The analogue sensor “Wearable Memory” was connected to display portal where photo content was displayed when residents approach the display. Family members could continually provide pictures for the device.	N/A	maintain family relationships, establish relationship with other residents	dyad, group	social isolation	---
Prophater et al.	2021	US	staff (*n* = 107)	User data, survey	Combined digital intervention	Intervention “Vital” includes providing a tablet with a tailored interface for residents as a videoconferencing tool to use with their families and a video-based learning platform with evidence-based dementia care training for staff and opening up a virtual forum to engage stakeholders	N/A	maintain family relationships	dyad, group	loneliness, social isolation	---
Rosa Hernandez et al.	2020	AU	residents (*n* = 12), staff (*n* = 3), informal caregivers (*n* = 10)	Observations, semistructured interviews	Intergenerational playgroup	Residents, children and their parents participated in an arranged playgroup involving free play for the children and a shared activity	N/A	establish intergenerational relationships	group	loneliness, social isolation	---
Theurer et al.	2020	CA	resident mentors (*n* = 48), resident mentees (*n* = 74), community-dwelling older adult mentors (*n* = 65), staff (*n* = 24)	Survey, assessment tools	Peer-mentorship programme	The “Java Mentorship” intervention engages community and resident volunteers as mentors. The mentors received education and provided visits to cognitively impaired resident mentees.	facilitate peer meetings, meaningful activity	establish long-term peer relationships	dyad, group	loneliness	Reported a decrease in loneliness.
Theurer et al.	2021	CA	resident mentees (*n* = 74)	Surveys, assessment tools, interviews	Peer-mentorship programme	The “Java Mentorship” intervention engages community and resident volunteers as mentors. The mentors received education and provided visits to cognitively impaired resident mentees.	facilitate peer meetings, meaningful activity	establish long-term peer relationships	dyad, group	loneliness, social isolation	Reported a decrease in loneliness and an increased desire to connect.
Theurer et al.	2022	CA	resident and community-dwelling mentors (*n* = 48)	interviews	Peer-mentorship programme	The “Java Mentorship” intervention engages community and resident volunteers as mentors. The mentors received education and provided visits to cognitively impaired resident mentees.	facilitate peer meetings, meaningful activity	establish long-term peer relationships	dyad, group	loneliness, social isolation	---
Tsai & Tsai	2011	TW	residents (*n* = 90)	Assessment tools	Laptop-based videoconferencing tool	Residents received a videoconference interaction with their family members.	Facilitate communication	maintain family relationships	dyad	loneliness	Reported a significant decrease in loneliness.
Tsai et al.	2020	TW	residents (*n* = 62)	Assessment tools	Smartphone-based videoconferencing tool	The intervention “Line” consists in a videoconferencing tool for residents to use with family members. Family members received conversational aids.	Create opportunities for interaction	maintain family relationships	dyad	loneliness	Reported a significant decrease in loneliness.
van Dyck et al.	2020	US	residents (*n* = 30)	Informal responses	Phone calls	The intervention consists in phone calls between residents and university student volunteers. The volunteers received conversational aids and instructions.	N/A	establish intergenerational relationships	dyad	loneliness, social isolation	---
Zamir et al.	2018	GB	residents (*n* = 19), hospital patients (*n* = 15), informal caregivers (*n* = 15), staff (*n* = 31)	Observations, interviews, feedback forms, reflexive diaries	Videoconferencing tool	“Skype on Wheels” is a tablet- and TV-based videoconferencing tool for residents to use with family members.	facilitate face-to-face contact	maintain family relationships	dyad	loneliness, social isolation	Reported improved perceived loneliness and social isolation only in nursing home residents.
Zamir et al.	2020	GB	residents (*n* = 22), staff (*n* = 8)	Observations, interviews, feedback forms, reflexive diaries	Videoconference quiz session	The “Intergroup Skype Quiz Sessions” via videoconferencing tool connected different nursing homes for games with fellow residents.	facilitate face-to-face contact	establish peer relationships	group	loneliness, social isolation	Reported increased socialization and situationally eased loneliness.
Zamir et al.	2021	GB	residents (*n* = 20), students (*n* = 6), teacher (*n* = 1), staff (*n* = 6)	Feedback forms, interviews, field notes	Videoconferencing tool	“Skype on Wheels” is a videoconferencing tool for residents to use with school students. The students received conversational aids.	Offer opportunity for socialisation	establish intergenerational relationships	group	loneliness, social isolation	Reported improved socialization.

### Study characteristics

The articles reported on interventions conducted in Canada (*n* = 11), the United Kingdom (*n* = 6), Australia (*n* = 5), the United States (*n* = 5), Taiwan (*n* = 2) and Germany (*n* = 1), with two studies not clarifying the country. Most texts were published between 2020 and 2022 (*n* = 22), while publications during the previous periods between 2011 and 2013 (*n* = 4), 2014 and 2016 (*n* = 2) as well as 2017 and 2019 (*n* = 5) remained relatively stable. Most studies employ a qualitative design (*n* = 12), while slightly fewer chose a mixed-method approach (*n* = 8). A smaller number of articles used a quantitative design (*n* = 5), did not clarify the data collection approach (*n* = 3) or did not gather empirical data at all (*n* = 5). Articles not based on empirical data included study protocols and concept proposals.

Residents were the main target group of all interventions, i.e. the interventions aimed at mitigating loneliness and social isolation in residents. Some interventions focused on residents with cognitive impairment only (*n* = 5), while most targeted older adults living in nursing homes in general (*n* = 28).

Most studies reported on interventions that introduced a digital technology or that trained a population in the usage of a digital technology (*n* = 17). Such interventions consisted of videoconferencing tools delivered through smartphones, computers, televisions or robots [34–40]; other communication technology, such as messaging apps, online platforms or e-mail programs [41–46]; or the technological delivery of entertainment activities or therapeutic support, i.e., video gaming and digital reminiscence tools [47–49] or a combination of various modified tablet functions [50].

Other studies reported introducing therapeutic or leisure activities into nursing homes (*n* = 11), such as music- and art-based activities [51–55], intergenerational visiting programmes [56–58] and peer-mentoring [59–61]. The remaining articles described changes in the management, living arrangements and philosophy of care at nursing homes (*n* = 5), including a multi-componential strategy [62], a plan on how to design and execute an integrated intervention [63], the Eden Alternative programme [64] and reports on intergenerational living arrangements [65, 66].

### Definition and measurement of loneliness and social isolation

Most texts only loosely referred to the concepts of loneliness and social isolation (*n* = 13); i.e., they did not define one or both concepts or systematically assess them as outcomes. A majority of studies does not report on loneliness or social isolation as an outcome (*n* = 16). The UCLA Loneliness Scale [67–69] was the most frequently used assessment tool for loneliness, but was used in different versions, while the de Jong–Gierveld Loneliness Scale [70] was only sporadically found. Only two studies reported on loneliness with qualitative interviews. The studies measured social isolation with the Duke Social Support Index [71], the Social Support Behaviours Scale [36] and the Friendship Scale [72].

In general, the term loneliness was used more frequently than social isolation. Most studies employ both terms (*n* = 19), while fewer use loneliness (*n* = 10) and only a fraction refer to social isolation (*n* = 4). There was a lack of consistency in how the two concepts were defined and, subsequently, how they were distinguished from each other. Instead, the articles revealed various interlinked concepts.

Although the articles did not unilaterally share a definition of loneliness and/or social isolation, in discussing these concepts, they mainly touched upon three dimensions. First, they referred to the quantity of social interactions. Thus, social isolation was seen as the objective lack of interactions [51], referring to the number of social contacts and interactions [53] or criticizing the lack of opportunities for nursing home residents to interact with each other or external actors [65]. The second dimension concerns the subjective perspective on interactions: Several studies claimed that loneliness occurs when individuals do not consider their existing interactions to be meaningful and fulfilling. For instance, two studies defined social connectedness as the existence of meaningful social interactions [41, 42]. This dimension includes the definition of loneliness as a misfit between the desired and the actual number of social interactions, i.e., the involuntary state of social isolation [44]. The third dimension sheds light on the biographical changes in social relationships and, subsequently, the distinction of primary and secondary informal caregivers. The studies hence argued that loneliness occurs when existing social relationships dissolve or when individuals find themselves incapable of establishing new social ties. The conceptual texts referred to the loss of social ties in older adults living in nursing homes in general [63] or specifically to the loss of friends and family members [62].

### Intervention mechanisms

Most studies did not sufficiently lay out the mechanism of the intervention. A large amount of studies did not explicitly mention how the intervention brings about reduced loneliness and/or social isolation (*n* = 12). In addition, in most cases the description of the mechanism was reduced to a singular sentence and did not give details about the interaction of elements of an intervention with these mechanisms. Comparing the interventions based on the three dimensions of loneliness and social isolation – the quantity of social interactions, the perception of these interactions and the biographical changes in social relationships – highlights three aspects of intervention mechanisms. These are not mutually exclusive and occur in various combinations in these studies.

The first aspect of intervention mechanisms is creating opportunities for social contact. Thus, the technologies, activities or large-scale changes in management were designed to decrease loneliness and social isolation by means of introducing or increasing encounters with informal caregivers. Several studies offered opportunities for interaction or connections (*n* = 8) or facilitated communication (*n* = 6). With regard to the quantity of interactions, the studies converged in establishing opportunities for increased encounters but diverged on how much time participants spent with others and how many actors were involved during the intervention. For example, one intervention involved 30 min of exchange per week [46], whereas another intervention involved 30 h of activities per month [66]. The way informal caregivers were involved also differed: The sample includes interventions involving informal caregivers in group activities as well as dyadic (i.e. person-to-person) encounters. There are interventions combining group and dyadic elements (*n* = 12), dyadic interventions (*n* = 10) and group interventions (*n* = 9).

The second aspect of ways to decrease loneliness and social isolation consists in creating meaningful connections. With regard to this, studies did not only increase or introduce contacts, the intervention was designed to foster meaningful exchange with informal caregivers. Several interventions were rooted in reflection on meaningful activities (*n* = 4) or concluded that an encounter is meaningful based on a proxy such as spontaneous activities, shared goals of participants or the length of interactions [42, 65]. However, the studies did not point out how tailored forms and durations of interactions were provided during the intervention to cater to the individual perceptions of the participants, which went beyond simple nonparticipation. One conceptual text therefore suggested that strategies to combat loneliness should include assessing loneliness scores, encouraging nursing staff to continuously monitor the quality of social interactions and designing a customized plan together with each resident [63].

Finally, the third aspect of intervention mechanisms consists in either establishing or maintaining social relationships. This aspect focusses on the informal caregivers involved and pinpoints the heterogeneity of people involved in nursing homes. For primary informal caregivers, the group most frequently addressed by these interventions was relatives (*n* = 12). In comparison, fewer studies mentioned friends (*n* = 2). For secondary informal caregivers, there were several intergenerational interventions involving children and their parents, schoolchildren and university students (*n* = 8) and interventions that addressed the potential of older adults and, in particular, other residents as informal caregivers (*n* = 8).

Most interventions either maintained contact with primary caregivers or created new social relationships with secondary caregivers, and only a few studies combined both aspects. In one intervention, trained student volunteers offered technical assistance to residents in sending emails to family members [45], a second intervention combined a customized tablet interface for families with a virtual stakeholder forum [50] and a third intervention displayed photographs uploaded by family members which could be seen by other residents and were to foster exchange between them [49]. The Eden Alternative established regular ties with children and other residents while also encouraging meaningful family connections [64], and another program encouraged peer support and the maintenance of contact with friends and family [62].

The interventions created opportunities for new groups to become involved in nursing homes. Visits included regular phone calls with university students [46] and video calls with schoolchildren [40]. Furthermore, a peer-mentoring programme arranged visits between community and resident mentors and cognitively impaired resident mentees [59–61]. Three studies described interventions establishing leisure activities, such as seasonal activities with university students [56], gardening, food preparation and related social events with schoolchildren [57]. There were several interventions that introduced games: an open playgroup with nursing home residents, younger children and their parents [58]; online quiz sessions [39]; and exergaming [47] with residents. Music-based interventions ranged from choir visits [54] to interactive music sessions with music experts [55] and multicomponent music approaches [51, 53]. Two studies evaluated intergenerational living arrangements, where students moved into a nursing home for a certain amount of time and fulfilled tasks for residents [65, 66].

Fewer interventions focused on maintaining existing relationships, and all of these interventions relied on digital technology. Videoconferencing was used to allow digital visits with friends and family members [36, 37], which were facilitated by a wheeled device [38] or a telepresence robot [34, 35]. Some interventions combined videoconferencing with asynchronous or text-based communication technologies. Examples include a multipurpose software using video, audio and picture content [41, 42]; and a platform combining video calls, voice-mail and text messaging [43]. There were online communication interventions [44] and, in particular, digital reminiscence applications that allowed primary informal caregivers to upload photos, music, books and video clips to a private device [48] or provide pictures that would automatically pop up on a public screen in the nursing home [49].

### Discussion

The review shows the breadth of interventions addressing loneliness and social isolation by involving different groups of informal caregivers in nursing homes. However, these studies rarely described explicitly how the intervention influenced or aimed at influencing loneliness and social isolation. Thus, as also pointed out in the latest update of the Medical Research Councils guidance on developing and evaluating complex interventions [30], there is a need for future intervention studies to give detailed descriptions on the mechanisms underlying interventions.

Only a limited amount of studies focused on residents with cognitive impairment and previous reviews have concluded that studies on interventions against loneliness and social isolation often exclude them [3, 29, 73]. Although there are already approaches to the inclusion of family members of residents with cognitive impairment [74], there is a need for further research on how these interventions can address loneliness and social isolation. Such interventions can profit from first in-depth insights on how these residents perceive loneliness [75].

### Three dimensions of loneliness and social isolation

The review revealed a plurality of assessment tools for and definitions of loneliness and social isolation in intervention studies. Several studies used the UCLA Loneliness Scale to report on loneliness scores – a trend emphasized in other reviews [3, 28, 29] – whereas there was no dominant assessment tool used to measure social isolation. In addition, other reviews have also highlighted the lack of coherence in defining these terms in intervention studies [5, 25]. As the studies did not provide such an explicit coherent use of definitions and measurements, we compared the definitions, measurements and implicit uses of loneliness and social isolation and thus highlighted three distinct dimensions in which the studies employed these concepts: the quantity of social interactions, the perception of these interactions and biographical changes in social relationships.

Analysing the quantitative dimension of loneliness and social isolation revealed that the most interventions converged in offering opportunities for social interactions, but covered different time frames and group sizes. The dimension of individual perception of social interactions corresponds to the cognitive theory of loneliness by Letitia Anne Peplau and Daniel Perlman defining loneliness as a discrepancy between actual and desired relationships [76]. Considering this dimension of loneliness in interventions goes beyond changing the amount of social contacts and highlights the need to assess the desired relationships of study participants. Although the studies all mentioned the perception of social relationships, this was not systematically evaluated during the delivery of the interventions. It is therefore not possible to deduce from the results of the studies which attributes of social relationships have an effect on social isolation and loneliness. Studies show, for example, that evenings and weekends are experienced as particularly lonely or that types of relationships play an important role and the meaning of loneliness for people is highly individual [75, 77]. Future studies should explicitly describe and evaluate these influencing elements.

Robert Weiss’s distinction between social loneliness, the absence of intimate relationships, and emotional loneliness, the lack of an overall sense of connectedness, captures the difference between maintaining the ties with primary informal caregivers and establishing new social relationships with secondary informal caregivers. Analysing the studies on the dimension of biographical changes in social relationships showed that informal caregivers are a heterogeneous group consisting of relatives and friends with established relationships with nursing homes residents as well as newly formed contacts spanning different age groups and organizational backgrounds such as schools, universities, or other nursing homes. Overall, the studies differed regarding the question which informal caregivers are most important for combatting loneliness and social isolation in nursing home residents. Other studies mainly point towards the role of family relationships in general [18] and the support of adult children in particular [17]. Some of the included studies targeted residents themselves as informal caregivers to their peers. Common activities between residents might increase overall social interactions [78]. Residents might especially be able to provide company and peer support to each if engaged in meaningful collective activities [16, 79]. Working with the distinction of social and emotional loneliness can especially shed light on the mechanisms of those interventions which combine meaningful activities with the creation of new social relationships [59–61].

### The mechanisms of interventions alleviating loneliness and social isolation

There is an overall need for studies to clarify the mechanisms through which interventions involving informal caregivers decrease levels of loneliness and social isolation, as pointed out for the case of befriending programmes [80]. The review highlighted three main aspects of the mechanisms through which interventions involving informal caregivers in nursing homes target loneliness and social isolation: Increasing social interactions, assuring meaningful and desired relationships and establishing new or maintaining existing relationships. Studies thus have to show, how an intervention sparks social interaction, how it ensures that these interactions are meaningful for and desired by the individuals participating in the implementation and how it relates to the biography of the target group.

The interventions included in this review ranged from short-term visits, to recurring weekly activities, to long-term live-in arrangements, and they included large groups as well as individual meetings. Earlier reviews classified interventions against loneliness, among other things, as person-to-person or group interventions [26, 27, 73, 81] and noted that in nursing homes, group interventions are more common than one-on-one approaches [73]. The review showed that interventions involving informal caregivers equally created group and dyadic settings or even combined both forms with each other.

Several interventions in this sample revolved around the questions of meaningful or purposeful activity, a tendency also highlighted in another review [26]. Although the studies emphasized individual perceptions of and needs for social interactions, they did not establish how they considered this aspect during the delivery of the interventions. There is first evidence on the impact of person-centred care on loneliness among nursing home residents [29] and the aspects of meaningful engagement [16, 82]. Therefore, there is a need to further consider multiple ways of participating in a single intervention and modes for adjusting the implementation to individual needs.

Interventions focus on maintaining social relationships established prior to residents moving into a nursing home or creating opportunities for new encounters for residents. While a review on interventions against loneliness in older adults reported on a dominance of approaches maintaining social relationships [26], both kinds of mechanisms are covered by intervention studies in this sample. However, it is possible that interventions establishing or maintaining social relationships might use different components for similar interventions. For example, it is unclear if a videoconferencing with family members and strangers needs the same or different supporting documents such as conversation aids.

### Limitations

The articles covered a limited geographical area, with many studies from Canada. In addition, the review was limited to recent articles, and thus, certain interventions – especially technological innovations – might have been overrepresented. Although the review covered a wide array of interventions, there are other interventions that might have the potential to include primary or secondary informal caregivers in the nursing home, such as those focused on religious and cultural practices, humour therapy, gender-based groups, animal-assisted therapy or befriending [3, 5, 26–29]. Conceptually, employing both social isolation and loneliness might have focused the perspective of this review more on the social aspects and less on the personality aspects of these phenomena [13]. Especially the existential dimension of loneliness lies beyond the scope of the interventions included in this review. The concept of involvement limited the search: The interventions we addressed involved primary and secondary informal caregivers in everyday activities and did not try to tackle loneliness and social isolation through occupational activities or therapeutic interventions alleviating maladaptive forms of cognition [26]. This limitation equally holds true for other aspects of primary informal caregiver involvement, such as being informed about care processes or participating in decision-making.

## Conclusion

The heterogeneity of definitions and measures of both loneliness and social isolation as well as the lack of sufficient descriptions of intervention mechanisms complicate the evaluation of interventions involving primary or secondary informal caregivers. When addressing the loneliness and/or social isolation in nursing home residents, it is necessary to clarify the dimension of loneliness and social isolation, e.g., the quantity of social interactions, the individual perspective on these encounters and the maintenance and establishment of social relationships. Furthermore, it has to be highlighted in what way the intervention and its components engage with these aspects. The interventions created opportunities for group and dyadic interactions, offered meaningful activities and brought various groups of informal caregivers into the nursing home through introducing technological advances, therapeutic and leisure activities or large-scale managerial changes or care philosophies to the nursing homes. There is especially a lack of evidence on the implementation of large-scale programs or care philosophies focused on the issue of loneliness and social isolation in nursing homes and interventions should increasingly offer multiple ways of participating to cater to person-specific requirements.

## Data Availability

All data generated or analysed during this study are included in this published article.
